# Preferences for HIV testing services among young people in Nigeria

**DOI:** 10.1186/s12913-019-4847-x

**Published:** 2019-12-27

**Authors:** Ucheoma Nwaozuru, Juliet Iwelunmor, Jason J. Ong, Sawsan Salah, Chisom Obiezu-Umeh, Oliver Ezechi, Joseph D. Tucker

**Affiliations:** 1 Saint Louis University College for Public Health and Social Justice, St. Louis, MO USA; 2 Central Clinical School, Monash University, Clayton, Australia; 3 Department of Global Health and Development, London School of Hygiene and Tropical Medicine, London, UK; 4 The Nigerian Institute of Medical Research, Yaba, Nigeria; 5 University of North Carolina at Chapel Hill, Chapel Hill, NC USA; 6 London School of Hygiene and Tropical Medicine, London, UK

**Keywords:** HIV testing, Preferences, Young people, Nigeria

## Abstract

**Background:**

Despite high HIV incidence rates among young people in Nigeria, less than 24% of this population have ever tested for HIV. These low HIV testing rates suggest that current testing services may not align with their testing preferences. To address this gap, the objective of this study was to assess preferences for HIV testing options among young people in Nigeria.

**Methods:**

We conducted a cross-sectional study using survey to assess preferences for HIV testing options among 113 youth aged 14–24 years residing in Nigeria. The survey included a series of hypothetical HIV testing options, comprised of six characteristics centered around HIV testing service (i.e. location of testing, test administrator, mode of pre-test, mode of post-test counseling, type of HIV test, and cost of HIV test). For each characteristic, participants were asked to select one of the options that they prefer or indicate none of the above. A descriptive analysis of the preferences made by participants was conducted, summarizing proportions of participants who selected different options for HIV testing.

**Results:**

The mean age of study participants was 19.5 years old (SD = 2.7). Most youth (73, 64.6%) had at least a secondary school degree. There was pronounced heterogeneity in HIV testing preferences among young people. Although most youth preferred free HIV testing, 14 (16.7%) reported preferring paying a small amount compared to free testing. More youth preferred blood-based HIV self-testing 39(48.8%) compared to facility-based HIV testing and oral HIV self-testing.

**Conclusions:**

Our finding suggest that young people have a range of HIV testing preferences in Nigeria. This suggests that a “one-size-fits-all” approach to delivering services to youth may be challenging in this context. HIV testing services can be optimized to reach young people if a variety options are provided to meet their unique preferences.

## Background

HIV incidence remains high among young people (i.e. age 14–24 years) in sub-Saharan African (SSA) countries despite decreases in HIV incidence among other groups [1]. In Nigeria, about 20% of new HIV infections occur among this age group [2, 3]. HIV testing is a critical component of comprehensive HIV services for youth [4]. However, among Nigerian youth, only about one-quarter have ever tested for HIV [4]. There are significant individual, structural, and social level barriers to HIV testing among Nigerian youth [5]. Anticipated HIV stigma and discrimination [5], lack of confidentiality, fear of testing outcomes partly explain this low uptake of HIV testing among young people in Nigeria [6–8]. These barriers undermine efforts to increase HIV testing and highlight the critical need to develop preference-sensitive, youth-centered and locally appropriate strategies.

Several studies have highlighted youth-centered and preference sensitive approaches as critical elements to promote uptake of health services among young people [9–12]. Specifically, these studies point out the importance of considering youth-preferences for health service delivery [9–12]. This is to ensure that services provided to young people are accessible, acceptable, affordable, equitable, appropriate and effective to address the needs of young people [12–14]. Youth-centered and preference sensitive services has shown promise in mitigating major barriers to health service uptake [9–12], specifically for health services that provide variety of options.

Regarding HIV testing, a range of options are currently available for young people to test for HIV. These testing options include location of testing (e.g. facility-based testing, home-based testing, and mobile testing), type of HIV test (e.g. oral HIV self-test, finger-prick HIV self-test, and health facility-based venipuncture blood test), and type of counseling (e.g. telephone counseling, one-on-one counseling, and handbook counseling) [15, 16]. Despite these various HIV testing options, research specifically focused on delineating characteristics of HIV testing services that may enhance HIV testing uptake among young people in Nigeria is lacking [17, 18]. To address this gap, the objective of this study was to identify youth preferences for various characteristics of HIV testing options in Nigeria.

## Methods

### Study area and participants

The study was conducted in Amuwo Odofin local government area in Lagos state, Nigeria among young people in 2018. Lagos State consists of 16 local government areas and has a population of about 21 million. Young people were defined as an individual between 14 and 24 years. Study participants were from a convenient sample recruited at a community youth health fair. The health fair was held at a community center and was attended by over 200 youth from Amuwo Odofin Local Government Area. The health fair consisted of interactive activities (such as: drunk booster goggles, virtual reality health games), informational booths on community health resources, mental health assessment and testing for HIV, STI, and blood pressure. Inclusion criteria for the study were: 1) young person aged between 14 and 24 years old, and 2) having provided informed consent.

### Data collection

We conducted a cross-sectional study using a self-administered written survey to assess preferences for HIV testing options among a sample of self-reported HIV-negative youth aged 14–24 years residing in Lagos state Nigeria. The survey was developed with a review of prior HIV testing literature in Nigeria and other SSA countries [5, 15, 16, 19–21]. The research team selected the icons to represent the HIV testing options and the developed survey was further pilot tested among 5 young people for their review and feedback. The survey was administered by two trained research assistants. All items on the survey were read aloud and explained to study participants for comprehension prior to survey completion as suggested by the individuals who pilot tested the surveys. Research assistants were available to respond to participants’ questions during survey completion. It took participants between 15 to 30 min to complete the survey. The survey was structured to include:

#### Section 1 (participants’ characteristics)

This contained questions on general sociodemographic characteristics (e.g. gender, age, marital status, religion, ethnicity and highest educational level), as well as sexual history (e.g. sexual activity status and age of sexual debut) [5, 19]. Participants age and age of sexual debut were reported as continuous variables, and others (marital status, religion, ethnicity, highest educational level, and sexual activity status) were categorical variables.

#### Section 2 (HIV prevention and awareness)

This contained questions on participants’ HIV testing history, knowledge of HIV self-test, risk perception of HIV and concerns for HIV [21]. These variables were reported as dichotomous variables: whether they had tested for HIV in their lifetime or not, whether had heard of HIV self-test or not, whether they thought they were at risk for HIV or not, and whether had concerns for HIV or not.

#### Section 3 (HIV testing options)

**Fig. 1:**
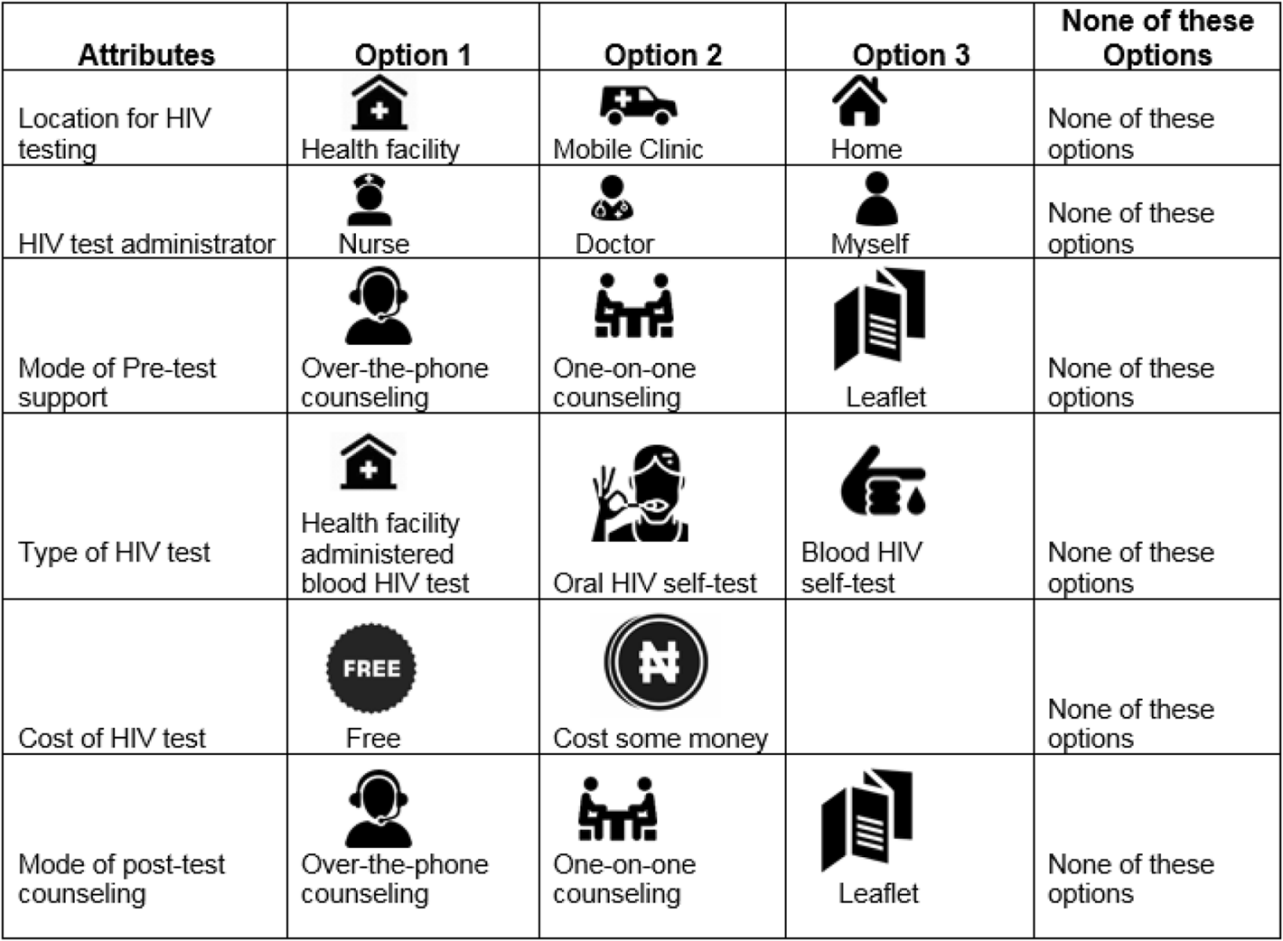
HIV testing service options provided in the survey

This contained a series of HIV testing options (i.e. location of testing, test administrator, mode of pre-test, mode of post-test counseling, type of HIV test, and cost of HIV test) [15, 16, 20]. For each characteristic, participants were asked to select one of the options that they prefer or indicate “none of the options provided”. The HIV testing options included in the survey are shown in Fig. 1.

### Statistical analysis

Participants’ demographic characteristics, as well as sexual and HIV testing history were summarized using descriptive analysis (frequencies, percentages, mean, and standard deviations). Preferences for the HIV testing options were described as proportions of their respective totals. Bivariate analyses (Fisher’s Exact test) was performed to assess the associations between selected demographic characteristics (i.e. gender, marital status, and highest educational level completed) and preferences for HIV testing options. Statistical Package for Social Sciences (SPSS) version 20 was used to input and analyze the data. Statistically significance was set at an alpha level of 0.05.

### Ethical approval

All study participants were provided with information on the study objective, as well as the potential benefits and risks, prior to participating in the study. All study participants provided informed consent before participating in the study. This study was approved by the Saint Louis University and the Nigerian Institute of Medical Research Institutional Review Boards.

## Results

### Participants characteristics

A total of 113 participants between the ages of 14–24 years completed the survey. Background characteristics of study participants are detailed in Table 1 and reflects representation of participants across sex, education, marital status, religion, ethnicity, and prior HIV testing. Most participants were women 84 (74.3%) and had completed at least secondary education 73 (64.6%). The mean age of study participants was 19.5 (SD = 2.7). About four-fifth (81.4%) of the participants reported being Christians and 68 (60.2%) were from the Igbo ethnic group. Fifty-four percent of the participants reported being sexually active with a mean age of sexual debut at 18 years.

**Table 1:** Selected characteristics of Nigerian youth, 2019 (*N* = 113)

Participants Characteristics	n (%)
Age, mean (SD)	19.5 (2.7)
Sex	
Female	84 (74.3%)
Male	22 (19.5%)
Not reported	7 (6.2%)
Highest level of education completed	
Secondary	73 (64.6%)
Technical Training	10 (8.8%)
Bachelors	25 (22.1%)
Not reported	5 (4.4%)
Marital Status	
Never married	109 (97.3%)
Married	1 (0.9%)
Not reported	3 (1.8%)
Religion	
Christian	92 (81.4%)
Muslim	17 (15.0%)
Other Religions	1 (0.9%)
Not reported	3 (2.7%)
Ethnicity	
Igbo	68 (60.2%)
Yoruba	34 (30.1%)
Hausa	4 (3.5%)
Other Ethnic groups	1 (0.9%)
Not reported	6 (5.3%)
Ever tested for HIV	28 (24.8%)

Data are number (percent) of participant, unless otherwise indicated

### HIV prevention and awareness

Twenty-eight (24.8%) of participants had ever tested for HIV in their lifetime and only 15 (14%) of the study participants had ever heard of HIV self-testing (HIVST). Only one study participant had ever used HIV self-testing. Additionally, 91 (87%) of the participants believed that they had no chance of being infected with HIV in the 3 months preceding the study. The majority of the participants 88 (83%) stated that they were concerned about HIV.

### Participants preferences for HIV testing service characteristics

**Fig. 2:**
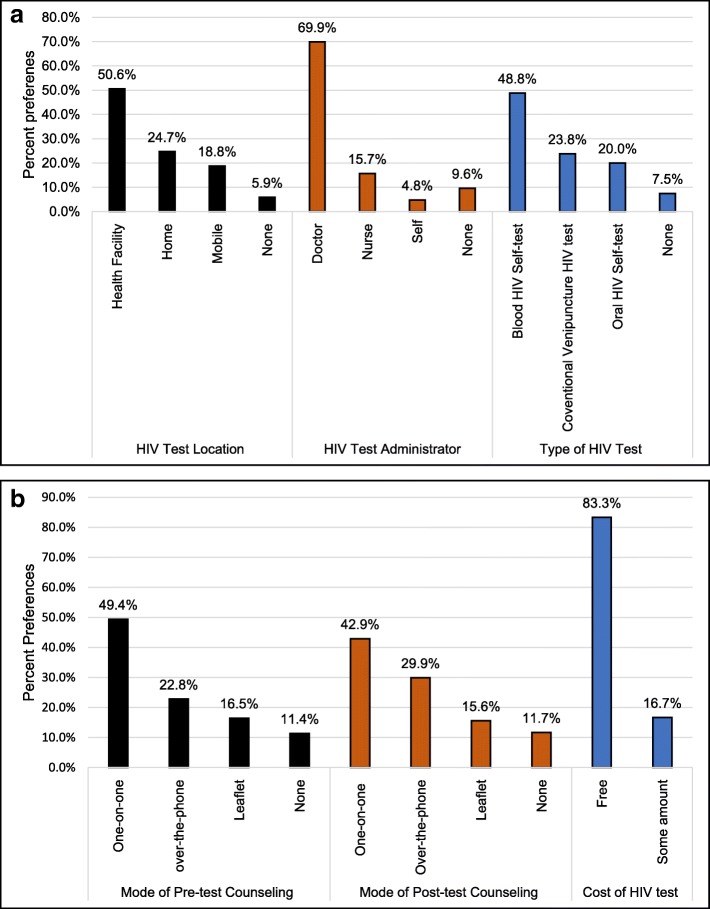
**a** Nigerian youth preferences for selected HIV testing services, 2018 (*N* = 113). Note: None indicates that participants selected “none of these options” in the choice survey. For some charts, the totals may differ from the participants’ totals owing to missing data. **b** Nigerian youth preferences for selected HIV testing services, 2018 (*N* = 113). Note: None indicates that participants selected “none of these options” in the choice survey. For some charts, the totals may differ from the participants’ totals owing to missing data

With respect to the location of HIV testing, most of the participants indicated preference for testing in a health facility 43 (50.6%), followed by home testing 21 (24.7%), and mobile testing 16 (18.8%). More than half of the participants 58 (69.9%) indicated that they would prefer a physician to administer the HIV test, followed by a nurse 13 (15.7%) and self-administered HIV test 4 (4.8%). For the type of HIV test, 39 (48.8%) of the study participants indicated that they preferred blood finger-prick HIV self-test, followed by conventional health facility administered venipuncture HIV test 19(23.8%) and oral HIV self-test 16(20.0%). Figure 2a shows participants’ preferences for location of HIV testing, HIV test administrator and type of HIV test.

For pre-HIV test counseling the majority of the participants preferred one-on-one testing 39(49.4%), followed by telephone counseling 18(22.8%), and informational leaflet 13(16.5%). For post-HIV test counseling, 33(42.9%) preferred one-on-one counseling, 23(29.9%) telephone counseling, and 12(15.6%) informational leaflet. Most participants 70(83.3%) indicated that they would prefer HIV testing services to be free. Figure 2b shows participants’ preferences for pre-and-post-test counseling and cost of HIV testing.

Overall, the majority of participants indicated preferences for three HIV testing options: 1) being tested by a doctor (69.9%), 2) having free HIV tests (83.3%), and 3) testing at a health facility (50.6%). Other important characteristics of HIV testing services included: using blood finger-prick HIV self-test (48.8%), having one-on-one pre-test counseling support (49.4%), and one-on-one posttest counseling support (42.9%).

### Indicators of participants preferences for HIV testing services

Bivariate analyses of participants’ sociodemographic characteristics and HIV testing service preferences revealed that gender is not statistically associated with participants’ preferences for any of the HIV testing options (*P* > 0.05). The results of the bivariate analysis are presented in Table 2. There was also no association between marital status and HIV testing option preferences, given that all but one of the participants were never married.

**Table 2:** The association between participants’ gender and HIV testing options, (*N* = 113)

	Female	Male	*P*-value
HIV testing location			0.46
Health facility	35 (44.3%)	6 (7.6%)	
Home	15 (19.0%)	4 (5.1%)	
Mobile	10 (12.7%)	4 (5.1%)	
None	3 (3.8%)	2.5%)	
HIV test administrator			0.72
Doctor	42 (54.5%)	12 (15.6%)	
Nurse	8 (10.4%)	3 (3.9%)	
Self	4 (5.2%)	0 (0.0%)	
None	6 (7.8%)	2 (2.6%)	
Type of HIV test			0.15
Blood HIV self-test	26 (35.1%)	8 (10.8%)	
Conventional Venipuncture HIV test	17 (23.0%)	1 (1.4%)	
Oral HIV self-test	10 (13.5%)	6 (8.1%)	
None	4 (5.4%)	2 (2.7%)	
Mode of Pre-test counseling			0.97
One-on-one	23 (31.5%)	10 (13.7%)	
Over-the-phone	15 (20.5%)	3 (4.1%)	
Leaflet	11 (15.1%)	2 (2.7%)	
None	7 (9.6%)	2 (2.7%)	
Mode of Post-test counseling			0.98
One-on-one	17 (23.9%)	10 (14.1%)	
Over-the-phone	19 (26.8%)	4 (5.6%)	
Leaflet	11 (15.5%)	1 (1.4%)	
None	7 (9.9%)	2 (2.8%)	
Cost of HIV test			0.52
Free	50 (64.1%)	14 (17.9%)	
Some amount	12 (15.4%)	2 (2.6%)	

Bivariate analysis between participant highest education level and preferences for HIV testing options shows a statistically significant association between preferences for cost of HIV test and highest education level. There was a minimally significant association between mode of post-test counseling and highest education level. These results are presented in Table 3. The bivariate analysis between religion and HIV testing options are provided in the Additional file 1: Table S1. There were no statistically significant association between religion and most of the HIV testing options. However, there is a statistically significant association between participants’ preference for the cost of HIV test and religion.

**Table 3:** The association between participants’ education level and HIV testing options, *N* = 113

	Secondary	Technical	Bachelors	P-value
HIV testing location				0.69
Health facility	32 (38.1%)	3 (3.6%)	7 (8.3%)	
Home	11 (13.1%)	2 (2.4%)	3 (3.6%)	
Mobile	15 (17.9%)	0 (0.0%)	6 (7.1%)	
None	4 (4.8%)	0 (0.0%)	1 (1.2%)	
HIV test administrator				0.47
Doctor	41 (50.0%)	4 (4.9%)	12 (14.6%)	
Nurse	10 (12.2%)	0 (0.0%)	3 (3.7%)	
Self	3 (3.7%)	1 (1.2%)	0 (0.0%)	
None	5 (6.1%)	0 (0.0%)	3 (3.7%)	
Type of HIV test				0.90
Blood HIV self-test	27 (34.2%)	2 (2.5%)	10 (12.7%)	
Conventional Venipuncture HIV test	14 (17.7%)	1 (1.3%)	4 (5.1%)	
Oral HIV self-test	10 (12.7%)	2 (2.5%)	3 (3.8%)	
None	5 (6.3%)	0 (0.0%)	1 (1.3%)	
Mode of Pre-test counseling				0.07
One-on-one	14 (17.9%)	2 (2.6%)	2 (2.6%)	
Over-the-phone	28 (35.9%)	0 (0.0%)	10 (12.8%)	
Leaflets	8 (10.3%)	3 (3.8%)	2 (2.6%)	
None	6 (7.7%)	0 (0.0%)	3 (3.8%)	
Mode of Post-test counseling				0.05
One-on-one	14 (18.4%)	1 (1.3%)	7 (9.2%)	
Over-the-phone	27 (35.5%)	0 (0.0%)	6 (7.9%)	
Leaflets	8 (10.5%)	3 (3.9%)	1 (1.3%)	
None	5 (6.6%)	1 (1.3%)	3 (3.9%)	
Cost of HIV test				0.03
Free	52 (62.7%)	2 (2.4%)	4 (18.1%)	
Some amount	8 (9.6%)	3 (3.6%)	3 (3.6%)	

## Discussion

One of the goals of the revised Nigerian National HIV/AIDS Strategic Framework is to increase demand and uptake of HIV testing among young people in Nigeria [2]. To achieve this goal, it is crucial to understand young people’s preferences for HIV testing options. This will inform developing preference-sensitive, and locally appropriate HIV testing services for young people in the country. This study was conducted to examine youth people’s preferences for different HIV testing options. Specifically, we were interested in understanding preferences for six HIV testing characteristics - location of testing, test administrator, mode of pre-test and post-test counseling, type of HIV test, and cost of HIV test. Overall, the results of the study show that young people have variabilities in their preferences for HIV testing characteristics. This study expands the literature by focusing on Nigerian youth, quantitatively examining HIV testing option preferences, and captured low-income youth.

First, our findings highlight heterogeneity in preferences for HIV testing services among Nigerian youth. These results are consistent with previous literature on young people’s preferences for HIV testing in Malawi, Zimbabwe and South Africa that suggest that one size does not fit all [15, 22]. Many youth preferred HIV self-testing services while others preferred facility-based testing. There were variations in participant’s preferences across the six HIV testing options provided to the participants. This suggests the need for diverse HIV testing service approaches for young people.

Second, most of the participants preferred blood-based HIV self-testing compared to conventional facility-based venipuncture HIV test. Young people’s preferences for blood based HIVST was surprising given the high preference for facility-based HIV testing. This discordance in preference for HIV testing could be because of limited knowledge and understanding of HIVST. Only a small proportion (14%) of the participants had ever heard of HIV self-testing and only one participant had used HIV self-testing. Nonetheless, this is congruent with other evidence which suggests preference for blood-based self-testing compared to facility-based venipuncture HIV test in sub-Saharan Africa [16, 23], Asia [24] and Europe [25]. Our findings also suggest that in addition to increasing knowledge of and awareness of HIV prevention, efforts should be made to integrate information on the full range of testing approaches and prevention tools currently available within Nigeria [2]. This is to ensure that young have people have improved access to and knowledge of HIV testing services in the country.

Third, most of the participants indicated preference for blood-based HIV self-testing compared to oral HIV self-testing. This is similar to the findings in Tanzania [26] and India [27], where participants indicated strong preference for finger prick HIVST compared to oral HIVST due to lack of familiarity and concerns of accuracy with oral HIVST. On the contrary, several other studies in SSA (Malawi, Zimbabwe, Mozambique and Kenya) [15, 28, 29] and the United States of America [30] have largely reported preference for oral HIVST compared to other conventional HIV testing and finger-prick HIVST among study participants. Oral HIVST was preferred because it was easy to use, does not require blood and painless [15, 28]. Given the focus of utilizing HIVST as an innovative and additional approach to increase HIVST among young people in SSA [2, 22], HIVST interventions or programs focused on increasing awareness and uptake of HIVST may need to address some of the concerns around HIVST while highlighting their benefits.

Some youth preferred paying a small amount for HIV testing compared to the majority who wanted HIV testing to be offered free of charge. Similar findings were reported in other studies among young people in SSA (Malawi and Zimbabwe) [15, 31, 32] where young people indicated preference for HIV testing to be free or very low cost due to high financial dependence of young people on their families. High cost attached to HIV testing acts as a barrier to HIV testing among young people [31]. In Nigeria, HIV test is done for free in government clinics and may also explain participants’ preference for free HIV testing [33]. While free HIV testing may not be sustainable for a country like Nigeria that is heavily reliant (95% of funding) on international donors for HIV prevention and management efforts [34], HIV preventions interventions should be cognizant of young people’s aversion to pay for HIV testing. HIV testing interventions or programs should be designed to provide low cost HIV testing options for young people. Also, we found statistically significant association between payment for HV test and some participants characteristics (education level and religion). This suggests that socio-demographic characteristics may influence preferences for paying for HIV tests. This association can be further explored in future studies to determine to what extent socio-demographic factors may explain young people’s preferences of HIV testing options such as payment for HIV test.

Not surprisingly, as other studies have clearly documented [31, 35], most the participants reported low or no risk perception for HIV. In our study, only 54 % of the participants were sexually active. This could explain the low risk perception among the participants. Nonetheless, it would be important to explore this further in future studies, as low HIV risk perception have been reported as a significant factor that hinder or limit uptake of HIV testing services among young people in other settings [31]. A study in Tanzania for example reported association between self-perceived risk of HIV and voluntary HIV testing and counseling [35]. In the study, participants who reported low self-perceived risk of HIV were less likely to test for HIV [35]. Thus, there is a need to tailor prevention messages correctly so that they reach young people in high seroprevalence settings in Nigeria, to increase uptake of HIV testing.

The limitations of this study should be kept in mind while interpreting its findings. One limitation is the potential sampling bias. Participants were conveniently recruited from a community youth health fair. Our study participants may be individuals who are already actively engaged towards improving their health. Nonetheless, the characteristics of the study participants are similar to other studies among young people Nigeria. These studies also recorded more female participants to male participants [36–39] and most of the young people had completed at least secondary school education [37, 40]. Another limitation is the social desirability response bias [41] or the possibility that some young people may have provided more socially acceptable responses, thus minimizing reporting their sexual and HIV testing history [42]. This was however mitigated by the anonymous nature of the self-administered survey. Finally, we did not confirm the HIV status of the study participants, which could potentially shape their preferences for HIV testing services [22].

The findings of this study have several implications for the design and implementation of HIV prevention programming, specifically preference-sensitive HIV testing options for young people in Nigeria. First, HIV prevention services should incorporate the needs and preferences of young people to enhance uptake HIV testing. Second, to achieve Nigeria’s HIV strategic objective of fostering an enabling environment where adolescents and young people have improved access to HIV testing services, our findings underscore the need to increase awareness of and access to newer HIV prevention services, including HIV testing options (e.g., free or reduced price or provider-initiated or self-tests). Efforts should also be made to identify ways to increase young people’s familiarity with these novel HIV prevention services to increase likelihood of uptake and/or consistent use. Equally important is working with young people themselves to better understand how and in what contexts these HIV prevention services may be adopted and consistently used [43, 44]. Thus, culturally appropriate interventions will be needed to engage young people in trying these unfamiliar HIV prevention tools. Data on the preferences of young people are also imperative to inform the design of youth-friendly interventions that are acceptable, accessible, and appropriate for all intended users.

## Conclusions

HIV testing is suboptimal among young people aged 14–24 years in Nigeria, yet few studies have examined this population’s preferences for HIV testing characteristics. Our findings suggest that young people have a range of preferences regarding HIV testing options in Nigeria. No single service is likely to be equally attractive or acceptable across different youth groups. Understanding young people’s preferences for HIV testing options is an important step toward promoting uptake of HIV testing among this population. These findings generate implications for policy makers and service providers that seek to create demand for and increase uptake of HIV testing among young people in Nigeria. The study results suggest that increasing awareness of and access to newer HIV testing options are necessary and may lead to increased uptake and adherence to prevention strategies that reduce HIV incidence in this underserved, vulnerable at-risk population. It also strengthens the call for further investigation into young people’s preferences to increase uptake of HIV testing services, including HIV self-testing.

## Supplementary information

**Additional file 1: Table S1.** The association between participants’ religion and HIV testing options.

## Data Availability

The datasets used for this study are available from the corresponding author upon reasonable request.

## Abbreviations

CO: Chisom Obiezu-Umeh
HIV: Human Immuno-deficiency virus
HIVST: HIV self-testing
JDT: Joseph D. Tucker
JI: Juliet Iwelunmor
JJO: Jason J. Ong
OE: Oliver Ezechi
SD: Standard deviation
SS: Sawsan Salah
SSA: Sub-Saharan Africa
UN: Ucheoma Nwaozuru

## References

[1] UNAIDS Data 2019 [https://www.unaids.org/en/resources/documents/2019/2019-UNAIDS-data].

[2] Revised National HIV and AIDS Strategic Framework 2019–2021:National Agency for the Control of AIDS.

[3] Nigeria: Country factsheets [https://www.unaids.org/en/regionscountries/countries/nigeria].

[4] Oginni AB, Adebajo SB, Ahonsi BA (2017). Trends and determinants of comprehensive knowledge of HIV among adolescents and young adults in Nigeria: 2003-2013. Afr J Reprod Health.

[5] Babalola S (2007). Readiness for HIV testing among young people in northern Nigeria: the roles of social norm and perceived stigma. AIDS Behav.

[6] Mathews C, Guttmacher SJ, Flisher AJ, Mtshizana YY, Nelson T, McCarthy J, Daries V (2009). The quality of HIV testing services for adolescents in Cape Town, South Africa: do adolescent-friendly services make a difference?. J Adolesc Health.

[7] Odo AN, Samuel ES, Nwagu EN, Nnamani PO, Atama CS (2018). Sexual and reproductive health services (SRHS) for adolescents in Enugu state, Nigeria: a mixed methods approach. BMC Health Serv Res.

[8] Young SD, Hlavka Z, Modiba P, Gray G, Van Rooyen H, Richter L, Szekeres G, Coates T (2010). Epidemiology and prevention HIV-related stigma, social norms, and HIV testing in Soweto and Vulindlela, South Africa: National Institutes of mental health project accept (HPTN 043). J Acquir Immune Defic Syndr.

[9] Ambresin A-E, Bennett K, Patton GC, Sanci LA, Sawyer SM (2013). Assessment of youth-friendly health care: a systematic review of indicators drawn from young people's perspectives. J Adolesc Health.

[10] Kurth AE, Lally MA, Choko AT, Inwani IW, Fortenberry JD (2015). HIV testing and linkage to services for youth. J Int AIDS Soc.

[11] Mazur A, Brindis CD, Decker MJ (2018). Assessing youth-friendly sexual and reproductive health services: a systematic review. BMC Health Serv Res.

[12] Erulkar Annabel S., Onoka Charles J., Phiri Alford (2005). What Is Youth-Friendly? Adolescents' Preferences for Reproductive Health Services in Kenya and Zimbabwe. African Journal of Reproductive Health.

[13] Organization WH (2002). Global consultation on adolescent friendly health services-a consensus statement.

[14] Barroso C (2014). Beyond Cairo: sexual and reproductive rights of young people in the new development agenda. Glob Public Health.

[15] Indravudh PP, Sibanda EL, d’Elbée M, Kumwenda MK, Ringwald B, Maringwa G, Simwinga M, Nyirenda LJ, Johnson CC, Hatzold K (2017). ‘I will choose when to test, where I want to test’: investigating young people’s preferences for HIV self-testing in Malawi and Zimbabwe. AIDS (London, England).

[16] Strauss M, George GL, Rhodes BD (2018). Determining preferences related to HIV counselling and testing services among high school learners in KwaZulu-Natal: a discrete choice experiment. AIDS Behav.

[17] Odimegwu CO, Imo CK, Amoo EO. HIV voluntary counselling and testing and behaviour changes among youths in Nigeria. J Biosoc Sci. 2019:1–16. 10.1017/S0021932019000506 31409439

[18] Oguegbu A, Beatty F (2016). Relationship between HIV counseling and testing (HCT) awareness and HCT uptake among Young people in Nigeria: implications for social change. World.

[19] Yahaya L, Jimoh A, Balogun O (2010). Factors hindering acceptance of HIV/AIDS voluntary counseling and testing (VCT) among youth in Kwara State, Nigeria. Afr J Reprod Health.

[20] Strauss M, George G, Mantell JE, Romo ML, Mwai E, Nyaga EN, Odhiambo JO, Govender K, Kelvin EA (2018). Stated and revealed preferences for HIV testing: can oral self-testing help to increase uptake amongst truck drivers in Kenya?. BMC Public Health.

[21] Oshi SN, Ezugwu FO, Oshi DC, Dimkpa U, Korie FC, Okperi BO (2007). Does self-perception of risk of HIV infection make the youth to reduce risky behaviour and seek voluntary counselling and testing services? A case study of Nigerian youth. J Soc Sci.

[22] Ritchwood TD, Selin A, Pettifor A, Lippman SA, Gilmore H, Kimaru L, Hove J, Wagner R, Twine R, Kahn K (2019). HIV self-testing: south African young adults’ recommendations for ease of use, test kit contents, accessibility, and supportive resources. BMC Public Health.

[23] Smith P, Wallace M, Bekker LG (2016). Adolescents’ experience of a rapid HIV self-testing device in youth-friendly clinic settings in Cape Town South Africa: a cross-sectional community based usability study. J Int AIDS Soc.

[24] Bien CH, Muessig KE, Lee R, Lo EJ, Yang LG, Yang B, Peeling RW, Tucker JD (2015). HIV and syphilis testing preferences among men who have sex with men in South China: a qualitative analysis to inform sexual health services. PLoS One.

[25] Witzel TC, Rodger AJ, Burns FM, Rhodes T, Weatherburn P (2016). HIV self-testing among men who have sex with men (MSM) in the UK: a qualitative study of barriers and facilitators, intervention preferences and perceived impacts. PLoS One.

[26] Ostermann J, Njau B, Brown DS, Mühlbacher A, Thielman N (2014). Heterogeneous HIV testing preferences in an urban setting in Tanzania: results from a discrete choice experiment. PLoS One.

[27] Sarkar A, Mburu G, Shivkumar PV, Sharma P, Campbell F, Behera J, Dargan R, Mishra SK, Mehra S (2016). Feasibility of supervised self-testing using an oral fluid-based HIV rapid testing method: a cross-sectional, mixed method study among pregnant women in rural India. J Int AIDS Soc.

[28] Ochako R, Vu L, Peterson K (2014). Insights into potential users and messaging for HIV oral self-test kits in Kenya, 3ie grantee final report.

[29] Hector J, Davies M-A, Dekker-Boersema J, Aly MM, Abdalad CCA, Langa EBR, Ehmer J, Hobbins MA, Jefferys LF (2018). Acceptability and performance of a directly assisted oral HIV self-testing intervention in adolescents in rural Mozambique. PLoS One.

[30] Merchant R, Clark M, Liu T, Rosenberger JG, Romanoff J, Bauermeister J, Mayer K (2017). Preferences for oral fluid rapid HIV self-testing among social media-using young black, Hispanic, and white men-who-have-sex-with-men (YMSM): implications for future interventions. Public Health.

[31] Peralta L, Deeds BG, Hipszer S, Ghalib K (2007). Barriers and facilitators to adolescent HIV testing. AIDS Patient Care STDs.

[32] Sibanda EL, d'Elbée M, Maringwa G, Ruhode N, Tumushime M, Madanhire C, Ong JJ, Indravudh P, Watadzaushe C, Johnson CC (2019). Applying user preferences to optimize the contribution of HIV self-testing to reaching the “first 90” target of UNAIDS fast-track strategy: results from discrete choice experiments in Zimbabwe. J Int AIDS Soc.

[33] Know your HIV/AIDS Status [https://naca.gov.ng/know-your-hivaids-status/].

[34] Donors provide 95% of HIV/AIDS funding in Nigeria –NACA DG [https://punchng.com/donors-provide-95-of-hivaids-funding-in-nigeria-naca-dg/].

[35] Wringe A, Isingo R, Urassa M, Maiseli G, Manyalla R, Changalucha J, Mngara J, Kalluvya S, Zaba B (2008). Uptake of HIV voluntary counselling and testing services in rural Tanzania: implications for effective HIV prevention and equitable access to treatment. Tropical Med Int Health.

[36] Thomas K, Fawole O, Al-ameen M (2015). HIV/AIDS voluntary counseling and testing (VCT): perspectives of rural youths in Oyo State, Nigeria. Int J Agric Econ Rural Dev.

[37] Somefun OD (2019). Religiosity and sexual abstinence among Nigerian youths: does parent religion matter?. BMC Public Health.

[38] Folayan MO, Harrison A, Brown B, Odetoyinbo M, Stockman JK, Ajuwon AJ, Caceres CF (2016). Associations between forced sexual initiation, HIV status, sexual risk behavior, life stressors, and coping strategies among adolescents in Nigeria. PLoS One.

[39] Ahonkhai AA, Banigbe B, Adeola J, Adegoke AB, Regan S, Bassett IV, Idigbe I, Losina E, Okonkwo P, Freedberg KA (2016). Age matters: increased risk of inconsistent HIV care and viremia among adolescents and young adults on antiretroviral therapy in Nigeria. J Adolesc Health.

[40] Agaba PA, Makai R, Bankat CT, Chebu PR, Apena T, Iyaji-Paul O, Idoko JA (2016). Sexual behavior and risk factors for HIV infection among young people aged 15-24 years in north-Central Nigeria. J Med Tropics.

[41] Latkin CA, Mai NVT, Ha TV, Sripaipan T, Zelaya C, Le Minh N, Morales G, Go VF (2016). Social desirability response bias and other factors that may influence self-reports of substance use and HIV risk behaviors: a qualitative study of drug users in Vietnam. AIDS Educ Prev.

[42] Moreno CL, EI-Bassel N, Morrill AC (2007). Heterosexual women of color and HIV risk: sexual risk factors for HIV among Latina and African American women. Women Health.

[43] Kubicek K, Arauz-Cuadra C, Kipke MD (2015). Attitudes and perceptions of biomedical HIV prevention methods: voices from young men who have sex with men. Arch Sex Behav.

[44] Hardee K, Gay J, Croce-Galis M, Afari-Dwamena NA (2014). What HIV programs work for adolescent girls?. J Acquir Immune Defic Syndr.

