# Supplementary material for: Preferences for HIV testing services among young people in Nigeria
Source: BMC Health Serv Res. 2019 Dec 27;19:1003. doi: 10.1186/s12913-019-4847-x (PMC6935128; doi:10.1186/s12913-019-4847-x)
Supplement: Supplementary file 1 — Additional file 1: Table S1. The association between participants’ religion and HIV testing options. [file 12913_2019_4847_MOESM1_ESM.docx]

**Table S1.** The association between participants’ religion and HIV testing options.

|  | Christianity | Islam | Other Religion | P-Value |
| --- | --- | --- | --- | --- |
| HIV testing location |  |  |  | 0.96 |
| Health facility | 35(42.2%) | 7(8.4%) | 1(1.2%) |  |
| Home | 17(20.5%) | 2(2.4%) | 0(0.0%) |  |
| Mobile | 14(16.9%) | 2(2.4%) | 0(0.0%) |  |
| None | 4(4.8%) | 1(1.2%) | 0(0.0%) |  |
| HIV test administrator |  |  |  | 0.51 |
| Doctor | 47(58.0%) | 10(12.3%) | 0(0.0%) |  |
| Nurse | 12(14.8%) | 0(0.0%) | 0(0.0%) |  |
| Self | 4(4.9%) | 0(0.0%) | 0(0.0%) |  |
| None | 6(7.4%) | 1(1.2%) | 1(1.2%) |  |
| Type of HIV test |  |  |  | 0.24 |
| Blood HIV self-test | 35(44.9%) | 3(3.8%) | 0(0.0%) |  |
| Conventional Venipuncture | 13(16.7%) | 5(6.4%) | 1(1.3%) |  |
| Oral HIV self-test | 14(17.9%) | 1(1.3%) | 0(0.0%) |  |
| None | 5(6.4%) | 1(1.3%) | 0(0.0%) |  |
| Mode of Pre-test counseling |  |  |  | 0.97 |
| One-on-one | 32(41.6%) | 5(6.5%) | 1(1.3%) |  |
| Over-the-phone | 16(20.8%) | 2(2.6%) | 0(0.0%) |  |
| Leaflets | 11(14.3%) | 1(1.3%) | 0(0.0%) |  |
| None | 8(10.4%) | 1(1.3%) | 0(0.0%) |  |
| Mode of Post-test counseling |  |  |  | 0.41 |
| One-on-one | 29(38.7%) | 3(4.0%) | 0(0.0%) |  |
| Over-the-phone | 20(26.7%) | 3(4.0%) | 0(0.0%) |  |
| Leaflets | 9(12.0%) | 1(1.3%) | 1(1.3%) |  |
| None | 8(10.7%) | 1(1.3%) | 0(0.0%) |  |
| Cost of HIV test |  |  |  | <0.01 |
| Free | 62(75.6%) | 6(7.3%) | 0(0.0%) |  |
| Some amount | 7(8.5%) | 6(7.3%) | 1(1.2%) |  |
